# Renewable Butanol Production via Catalytic Routes

**DOI:** 10.3390/ijerph182211749

**Published:** 2021-11-09

**Authors:** Heeyoung Choi, Jeehoon Han, Jechan Lee

**Affiliations:** 1 Department of Environmental and Safety Engineering, Ajou University, Suwon 16499, Korea; chk6788@ajou.ac.kr; 2 School of Semiconductor and Chemical Engineering & School of Chemical Engineering, Jeonbuk National University, Jeonju 54896, Korea; 3 Department of Energy Systems Research, Ajou University, Suwon 16499, Korea

**Keywords:** biomass, butanol, catalysis, organic waste, renewable chemical, sustainable chemistry

## Abstract

Fluctuating crude oil price and global environmental problems such as global warming and climate change lead to growing demand for the production of renewable chemicals as petrochemical substitutes. Butanol is a nonpolar alcohol that is used in a large variety of consumer products and as an important industrial intermediate. Thus, the production of butanol from renewable resources (e.g., biomass and organic waste) has gained a great deal of attention from researchers. Although typical renewable butanol is produced via a fermentative route (i.e., acetone-butanol-ethanol (ABE) fermentation of biomass-derived sugars), the fermentative butanol production has disadvantages such as a low yield of butanol and the formation of byproducts, such as acetone and ethanol. To avoid the drawbacks, the production of renewable butanol via non-fermentative catalytic routes has been recently proposed. This review is aimed at providing an overview on three different emerging and promising catalytic routes from biomass/organic waste-derived chemicals to butanol. The first route involves the conversion of ethanol into butanol over metal and oxide catalysts. Volatile fatty acid can be a raw chemical for the production of butanol using porous materials and metal catalysts. In addition, biomass-derived syngas can be transformed to butanol on non-noble metal catalysts promoted by alkali metals. The prospect of catalytic renewable butanol production is also discussed.

## 1. Introduction

The employment of fossil hydrocarbons has fostered population growth and prosperity, which has made our society heavily depend on fossil fuels and petroleum-derived chemicals. Recently, the demand for renewable fuels and chemicals has been increasing, associated with recent emphasis on less carbon-intensive options driven by environmental, social, and governance-minded investing strategies. The market size of renewable bio-based chemicals was forecast to be 106 billion USD with a compound annual growth rate (CAGR) of 10.6% from 2020 to 2025 [1].

Butanol, a four-carbon alcohol, is a commodity organic chemical that has a wide range of applications in manufacturing (polymers, synthetic rubber, brake fluids, lubricants, etc.), pharmaceutical, and cosmetics industries. It has been employed not only as an industrial solvent but also as an intermediate to produce vital chemicals such as acetates, acrylate esters, amines, amino resins, butyl acrylate, glycol ether, and methacrylate [2]. The global butanol market was valued at approximately 7 billion USD in 2020, expected to reach approximately 9 billion USD by 2026 with a CAGR of 3.7% from 2021 to 2026 [3]. Current commercial production of butanol is based on petroleum-derived chemicals. However, the petrochemical production of butanol is susceptible to the price of crude oil that fluctuates considerably. In addition to fluctuating crude oil price, the depletion of fossil fuel resources and the serious global environmental issue, climate change caused by global warming, have spurred the use of renewable sources (biomass, organic waste, etc.) as the feedstock for the production of commodity chemicals such as butanol.

The use of biofuels is crucial to reduce the use of fossil fuels. There is stimulating demand for biofuels, estimated to reach approximately 51 billion gallons per year by 2022 [4]. Although ethanol has been the most used biofuel, butanol (mostly n-butanol) is considered a better source of alternative fuel than ethanol because of its about 1.5 times higher energy density than ethanol [5]. Butanol is also less volatile, less corrosive, and less hygroscopic than ethanol, not only resulting in fewer ignition problems in engines but also allowing a safer engine operation without any modification [6,7,8] In addition, the combustion of butanol leads to a lower CO_2_ emission than that of ethanol and even gasoline [9], with no emission of nitrogen oxides and sulfur [10]. In this regard, the demand for the renewable butanol production is stimulating.

Biological production of butanol is considered a carbon-neutral pathway from biomass to butanol (i.e., biobutanol). Acetone–butanol–ethanol (ABE) fermentation, a process that employs bacterial fermentation using *Clostridium* spp. to produce acetone, n-butanol, and ethanol from carbohydrates such as starch and glucose, is the most widely studied method to convert biomass into butanol [11]. Nevertheless, ABE fermentation for biobutanol production still confronts significant challenges. The process results in byproducts, such as acetone, ethanol, 2-propanol, milk acid, and propanoic acid, which cause not only a decrease in the n-butanol yield but also an increase downstream processing cost for the purification of n-butanol [12]. In addition, the native microorganisms used for ABE fermentation (*Clostridium* spp.) suffer from solvent toxicity, complicated nutrient requirements, slow growth, and complex life cycle (i.e., the production of spores in *Clostridium*) [13,14]. It is also challenging to modify native producer strains using different genetic and synthetic biological methods [15]. High feedstock cost and high water usage are other challenges for ABE fermentation that hinder large scale production of butanol via the biological route [14]. The drawbacks of the fermentative biobutanol production highly necessitates the production of butanol from renewable resources (e.g., biomass) via nonfermentation routes.

Catalytic production of butanol from biomass-derived compounds has recently attracted great attention as an alternate route to synthesize renewable butanol. The primary advantage of catalytic routes is that they involve simple steps to achieve relatively high yields of butanol compared with fermentative route [16]. Given recent scientific research output, this review provides an up-to-date summary of knowledge of the catalytic conversion of biomass-derived compounds into butanol. We discuss catalysts investigated to transform different biomass-derived chemicals into butanol outlining various catalytic routes from the biomass compounds to butanol. Present challenges and perspectives of the catalytic production of renewable butanol are also discussed.

## 2. Butanol Production from Ethanol

**Figure 1:**
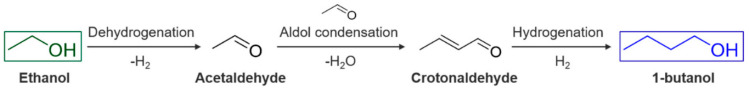
Representative reaction pathway for the conversion of ethanol to n-butanol via aldol condensation. Reprinted from Xi et al. [28] and licensed under CC BY 4.0.

As is well known, a large volume of ethanol made from renewable carbon-neutral resources (i.e., biomass), called bioethanol, is being employed not only as a biofuel additive for gasoline [17,18] but also as a feedstock of various chemicals [19,20,21,22]. Approximately 98.4 billion liters of bioethanol was produced in 2018 [23]; however, its water solubility, corrosivity, and the differences in fuel properties between bioethanol and conventional transportation fuels (e.g., gasoline) make it unsuitable to employ ethanol fuel in modern internal combustion engines [24]. Hence, the use of ethanol as the feedstock for the production of butanol has great potential. The process that converts ethanol to n-butanol is industrialized, which increases the carbon number by coupling two ethanol molecules. The Guerbet reaction is an aldol-condensation-type reaction of coupling alcohols, involving oxidation of alcohol to aldehyde, aldol condensation of the aldehyde to allyl aldehyde, and hydrogenation of the allyl aldehyde to its corresponding alcohol [25]. For the reaction from ethanol to n-butanol, dehydrogenation of ethanol occurs first to make acetaldehyde [26]. Aldol condensation of acetaldehyde to crotonaldehyde then takes place, followed by hydrogenation to form n-butanol [27]. The conversion of ethanol to n-butanol is described in Figure 1.

Various homogeneous and heterogeneous catalysts are available for the Guerbet reaction. For the homogeneously-catalyzed Guerbet reaction (taking place at 150–160 °C), Ru- or Mn-based homogeneous catalysts have been reported [29,30,31,32,33,34,35]. Despite high ethanol selectivity (>90%), the homogeneous Guerbet process leads to undesirable byproducts such as C_6+_ alcohols and sodium acetate [36]. In addition, the separation of catalyst from the reaction product (i.e., butanol) is another issue of the homogeneous reaction [37,38].

To avoid such problems, various heterogeneous catalyst systems that allow direct conversion of ethanol to n-butanol have been suggested [39,40,41,42,43,44]. Solid acid–base catalysts have been tested. For instance, a commercially available hydroxyapatite catalyst led to about 50% selectivity toward butanol at 350–440 °C with an indication of the formation of byproducts such as H_2_ and acetaldehyde [45]. Kozlowski and Davis experimentally proved that an increase in the density of base sites on a ZrO_2_ catalyst by an addition of 1 wt.% Na enhances the selectivity toward n-butanol because the dehydration of ethanol is significantly inhibited [46].

Ogo et al. synthesized four catalysts (Ca–P, Ca–V, Sr–P, and Sr–V) used for ethanol conversion to n-butanol [47]. The ethanol conversion reaction was conducted at 300 °C with a space velocity of 130 h g_cat._ mol_ethanol_^−1^. The Sr–P catalyst provided the highest selectivity toward n-butanol amongst the catalysts tested. This was because the Sr–P catalyst not only provided a high selectivity toward crotonaldehyde resulting from aldol condensation of acetaldehyde (an intermediate of n-butanol; Figure 1) but also inhibited the coke formation occurring in the hydrogen transfer reaction of 2-buten-1-ol into n-butanol. As the molar ratio of Sr/P became higher, the selectivity toward n-butanol was enhanced [48]. The density of strong acid and base sites increased as the molar Sr/P ratio increased, and the base site density was much higher than the acid site density. Aldol condensation was expedited by base catalysis; thus, the Sr–P catalyst having a higher base site density resulted in a higher catalytic activity for the production of 1-butanol from ethanol.

Catalysts consisting of Mg and Al have been investigated as an effective catalytic system for the selective conversion of ethanol to n-butanol. For example, modified MgO catalyst at 450 °C and 1 atm could transform ethanol into n-butanol [49]. It was proposed that the mechanism of the ethanol conversion over the MgO catalyst is similar to the case of basic zeolite: the basic metal oxide activates the C–H bond in β-position of ethanol followed by its condensation with another ethanol molecule via dehydration, resulting in the formation of n-butanol [50]. Other acid–base catalysts, e.g., Mg–Al mixed oxides, were investigated in a one-pot conversion of ethanol to n-butanol [51]. It was shown that a Mg–Al mixed oxide with a Mg/Al ratio of 3 led to ~38% selectivity toward n-butanol at an ethanol conversion of ~35% at 350 °C under 1 atm. The catalyst characterization results proved that adjacent acid and medium basic sites (Al inserted in MgO lattice or Mg inserted in Al_2_O_3_ lattice) promote the formation of butanol, because the presence of both sites is needed to form the reaction intermediate species. The Mg–Al oxide with a Mg/Al ratio of 3:1 led to a faster formation of intermediates to n-butanol (i.e., acetaldehyde and crotonaldehyde) than the Mg–Al oxide with a Mg/Al ratio of 1:1 [52]. This was due to fewer surface carboxylate functionalities on the Mg–Al oxide (Mg/Al = 3/1) than the Mg–Al oxide (Mg/Al = 1/1), given that the carboxylate functionalities compete with the catalytic active sites. León et al. also showed that higher concentration and strength of basic sites on Mg–Al mixed oxide catalyst resulted in higher selectivity toward C_4_ fractions for the ethanol conversion, while the presence of acid sites on the Mg–Al mixed oxide catalyst decreased the activity for condensation reaction by promoting ethanol dehydration [53]. The water might be continuously removed from the reaction mixture in order to further enhance the selectivity toward n-butanol [54].

**Figure 2:**
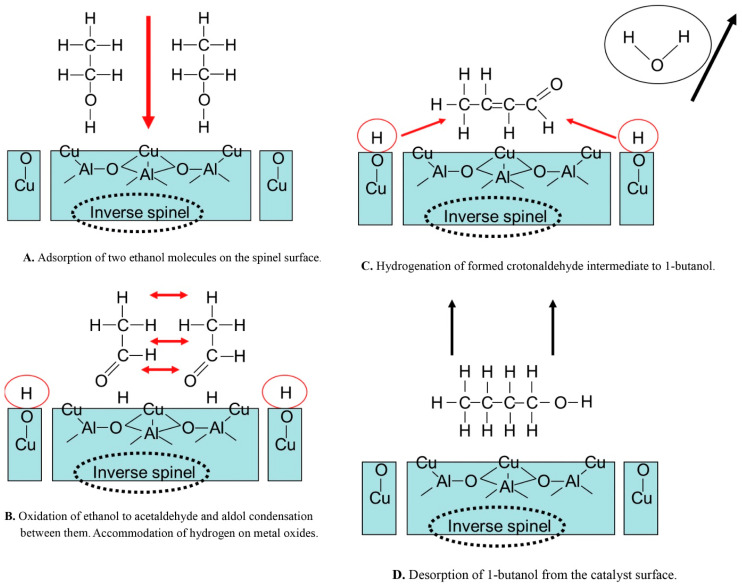
Proposed ethanol conversion mechanism for the production of n-butanol on alumina-supported metal catalysts at 240 °C under 7 MPa. Reprinted from Riittonen et al. [58], Copyright (2014), with permission from Elsevier.

Other than the aforementioned solid acid–base catalysts, the butanol production from ethanol over metal catalysts has been reported. Riittonen et al. screened a variety of supported metal catalysts (Pt, Ru, Rh, Ag, Au, and Ni) to find active catalysts for the direct conversion of ethanol to n-butanol [55]. The selectivity towards n-butanol followed an order of Ni > Pt > Rh~Au > Ru >> Ag. It was found that an Al_2_O_3_-supported Ni catalysts with a Ni loading of 20.7% was the most selective catalyst for the n-butanol production via dimerization of ethanol (80% selectivity at 25% ethanol conversion at 250 °C). A process that continuously converts ethanol to n-butanol using Ni/γ-Al_2_O_3_ catalyst was developed by Ghaziaskar and Xu [56]. The reaction was carried out in a continuous-flow packed-bed reactor at a range of temperature between 135 °C and 300 °C at a weight hourly space velocity (WHSV) between 6.4 h^−1^ and 15.6 h^−1^. The highest selectivity toward n-butanol (62% at 35% ethanol conversion) was achieved with an 8% Ni/γ-Al_2_O_3_ catalyst (stable for 18-h time-on-stream) at 250 °C under 17.6 MPa. Dowson et al. used Ru-based homogeneous catalysts to transform ethanol to n-butanol [57]. A 94% n-butanol selectivity at >20% ethanol conversion was obtained. It was suggested that the homogeneous catalytic system tamed uncontrolled base-catalyzed aldol condensation of acetaldehyde. Figure 2 shows a schematic of a mechanism of ethanol conversion to n-butanol over alumina-supported metal catalysts [58]. It consists of adsorption of two ethanol molecules on the spinel surface (Figure 2A), oxidation of ethanol to acetaldehyde and aldol condensation between these compounds with the accommodation of hydrogen on metal oxide (Figure 2B), hydrogenation of crotonaldehyde to n-butanol (Figure 2C), and desorption of n-butanol from the catalyst surface (Figure 2D).

The aforementioned studies into the ethanol conversion to butanol emphasized evaluating bulk catalytic materials. The studies to use nanosized catalysts for the ethanol-to-butanol reaction are very limited. The employment of nanocatalysts may enhance the conversion of ethanol and the selectivity toward butanol, considering that they are shown to be more active, selective, and stable than bulk-structured catalysts for various ethanol conversion reactions [59,60,61]. For example, Ce–La catalysts with a range of particle size between 1 nm and 10 nm required a lower temperature to obtain 100% ethanol conversion and had a greater stability (stable for 15 h time-on-stream without deactivation) than bulk catalytic materials [62]. Au nanoparticles (3–5 nm) dispersed on SiO_2_ were an active stable catalyst to oxidize ethanol to acetaldehyde, acetic acid, and acetyl acetate (37–58% yield) at 210 °C [63]. A nanosized HZSM-5 zeolite (~30 nm) showed higher activity and selectivity toward gasoline-range hydrocarbons than a bulk HZSM-5 zeolite [64]. The nano-HZSM-5 catalyst had 74% selectivity with a research octane number (RON) of 91 at 450 °C, while the bulk HZSM-5 catalyst had 59% selectivity with a RON of 87.

In Table 1, a wide range of catalysts and relevant reaction conditions for the conversion of ethanol to n-butanol are summarized, based on the results reported in earlier literature available.

## 3. Butanol Production from Butyric Acid

Ethanol production via fermentation of organic waste such as sludge, manure, and food waste is much more difficult than typical bioethanol production (i.e., ethanol from corn or sugarcane). The fermentative production of ethanol from organic waste involves the extraction of fermentable sugars from raw food waste through a series of pretreatment and hydrolysis steps. Due to the high moisture content and heterogeneously variable composition of food wastes, these processes are costly, leave behind a significant amount of residual waste, and can produce compounds that may inhibit microorganism activity during fermentation [67]. To avoid these problems, anaerobic digestion is commonly used to treat organic waste. In this process, monomers such as sugars, amino acids, and long-chain fatty acids are produced from complex polymers contained in food waste using hydrolytic bacteria. The monomers are then converted to volatile fatty acids (VFAs) through a series of acidogenesis and acetogenesis steps [68,69]. VFAs are carboxylic acids with less than C_6_ (e.g., acetic acid, propionic acid, butyric acid, isobutyric acid, valeric acid, and isovaleric acid). VFAs are the major intermediate in producing biogas by methanogenesis. Approximately 5.3 × 10^5^ tons of VFA generation in Republic of Korea was estimated [70].

**Figure 3:**
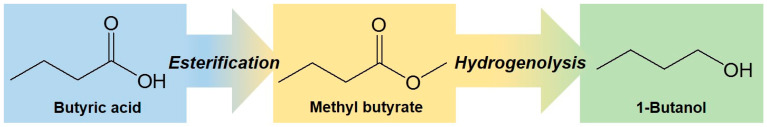
Strategic two-step process for the production of n-butanol from butyric acid. Reprinted from Cho et al. [75], Copyright (2019), with permission from Elsevier.

Among different VFAs, butyric acid, which is one of the most dominant in the product produced via anaerobic digestion of organic waste [71,72,73], has been considered a renewable feedstock for the production of butanol. A conversion route to produce butanol from butyric acid was suggested as a strategy for effective energy recovery from organic waste as a form of liquid fuel [74]. The two-step conversion pathway of butyric acid to butanol is shown in Figure 3 [75]. This pathway involves the production of butyric acid generated by anaerobic digestion of organic waste, which is then upgraded to butanol. The upgrading takes place via two steps: (1) esterification of butyric acid into methyl butyrate and (2) hydrogenolysis of methyl butyrate into butanol.

For the first step of the butyric acid-to-butanol pathway (i.e., esterification of butyric acid to methyl butyrate; Figure 3), researchers have recently demonstrated that porous carbon materials such as carbon nanotubes (CNTs) are an effective catalyst; ~90% yield was obtained at 360 °C under 1 atm (initial pressure) with an butyric acid/methanol volumetric ratio of 0.5 [76]. Table 2 lists other catalytic systems used for the esterification of butyric acid to methyl butyrate.

The esterification of butyric acid to methyl butyrate was found to occur inside the pores of a porous material. To initiate the reaction, the pore size of the porous material needs to be bigger than the kinetic diameter of butyric acid and methanol, which allows the collision between the two reactants [76]. In addition to pore size, pore geometry is also crucial. Hollow-rod-like open pores allow the reactants to readily enter the pores and the product to regularly exit the pores [76]. The surface functionality of the porous material also plays a key role in expediting the esterification reaction, which acts as catalytic sites that activate butyric acid and methanol [77].

For the second step of the butyric acid-to-butanol pathway (Figure 3B), a bimetallic Pt–Co catalyst was proven to be effective at hydrogenolysis of methyl butyrate to n-butanol [75]. A 54.1% selectivity toward n-butanol was achieved with a Pt–Co catalyst (Co/Pt molar ratio: 20) at 250 °C under 5 MPa H_2_. At above 250 °C and under above 5 MPa H_2_, the catalytic activity for the hydrogenolysis of methyl butyrate was deteriorated, leading to a lower selectivity toward n-butanol than that achieved at 250 °C under 5 MPa H_2_. The density-functional theory calculations showed that the activity for the methyl butyrate hydrogenolysis is correlated with the combined adsorption energy of methoxy and butyryl groups. Other catalysts employed for the production of n-butanol via hydrogenolysis are listed in Table 3.

For the hydrogenolysis of methyl butyrate to n-butanol, methane, methanol, butyl butyrate, and butyric acid were produced as byproducts [70]. Hydrogenolysis of methyl butyrate resulted either in n-butanol and methanol or in butyric acid and methane. Butyl butyrate resulted from transesterification of methyl butyrate and n-butanol. Density functional theory (DFT) calculation showed that the adsorption energy of methoxy and butyryl groups of the reactant molecules on the metal catalyst surface is highly associated with the methyl butyrate hydrogenolysis activity [75].

## 4. Butanol Production from Biomass Syngas

**Figure 4:**
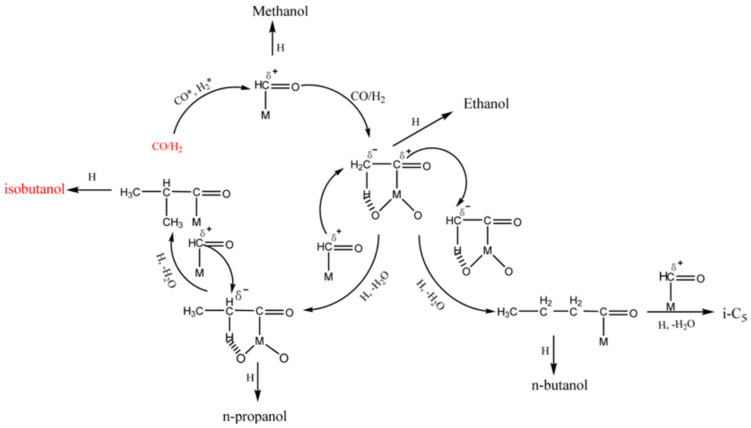
Reaction pathway for the formation of isobutanol from syngas. Reprinted from Wu et al. [82], Copyright (2014), with permission from Elsevier.

Gasification is a process to make synthesis gas (syngas), primarily composed of H_2_ and CO, from biomass. Syngas can be used to synthesize ammonia, methanol, and hydrogen. Recently, biomass syngas has been suggested as the feedstock for the production of isobutanol. The isobutanol production mechanism involves three-step consecutive reactions. The first reaction leads to the formation of methanol, the second reaction is to make ethanol, and the third reaction is to transform ethanol to isobutanol [81,82]. The addition of formyl intermediate to α-carbon in methanol forms ethanol. Aldol condensation of ethanol with methanol makes propanol, and the propanol is reacted with methanol to make isobutanol. It is difficult for isobutanol to undergo aldol condensation due to the lack of two β-hydrogens needed for its aldol condensation and its steric hindrance; thus, isobutanol is an end product of the chain growth reaction of alcohols. The syngas-to-isobutanol reaction is highly associated with reaction temperature. At low temperatures, linear chain growth taking place via CO insertion is dominant [83,84,85]. At high temperatures, however, branched chain growth reaction occurs through aldol-condensation of the linear alcohols produced at low temperatures [82]. Branched alcohols, such as isobutanol, are the primary products. Figure 4 schematically depicts the formation of isobutanol from syngas.

Non-precious metal catalysts modified with alkali metal promoter, such as Zn–Cr-based catalysts modified with K, were reported for the production of isobutanol from syngas owing to high activity and stability [86,87,88]. The catalysts had high CO conversion (e.g., 10–30%) and selectivity toward isobutanol (e.g., 20%). This does not necessitate a complex separation process that isolates butanol from the product stream. The effect of various alkali metals modifying the Cr/ZnO catalyst on the conversion of syngas to isobutanol was investigated, showing that a K-modified Cr/ZnO catalyst had excellent catalytic performance to produce isobutanol from syngas [89]. For the K-modified Zn–Cr catalyst, the non-stoichiometric spinel is the active phase for the conversion of syngas to isobutanol [88]. Textual properties, reducibility, and base density of the catalyst are also crucial for the production of isobutanol from biomass syngas.

## 5. Economic Approaches for the Catalytic Production of Renewable Butanol

There are a few studies with an economic assessment of the aforementioned catalytic processes, which deserve to be introduced in this review. Carmona-Garcia et al. compared the economic potential of two biobutanol production processes, such as ABE fermentation and catalytic upgrading of bioethanol [90] (refer Section 2). Given a daily processing scale of over 1000 tons, ABE fermentation provides a higher butanol production (2.59 tons per hour) with a lower net energy consumption (57.9 GJ per ton of butanol) than the catalytic ethanol upgrading. The minimum selling price of biobutanol was estimated to be 1.56 USD per kg of biobutanol (for ABE fermentation) and 1.80 USD per kg of biobutanol (for catalytic ethanol upgrading).

Kwon and co-workers performed technoeconomic analysis of the two-step butanol production from butyric acid shown in Figure 3 [75] (refer to Section 3). The minimum selling price of n-butanol produced from butyric acid via the two-step process (overall 48% yield of butanol from butyric acid; energy efficiency of 30%) was estimated to be 3.4 USD per gallon of gasoline equivalent, which falls within the recent biofuel market prices between 2.0 USD per gallon of gasoline equivalent to 3.8 USD per gallon of gasoline equivalent.

Okoli and Adams developed a model of the catalytic process from lignocellulosic biomass to butanol via syngas [91] (refer Section 4). With the assumption that the crude oil price is over 92 USD per barrel, the minimum selling price of butanol was determined as 0.8 USD per liter of butanol that could compete with the price of petroleum-derived butanol higher than 85.4 USD per barrel of gasoline equivalent. Feedstock costs, coproduct (mixed alcohols) value, and internal rate of return are significant factors that affect the economic viability of the syngas-to-butanol process.

## 6. Conclusions

Butanol from renewable resources such as biomass offers high potential not only for being an alternative to petrol but also being a feedstock for the production of other commodity chemicals. Catalytic routes from biomass-derived compounds to butanol could be classified into (1) ethanol to butanol via chain growth reactions; (2) butyric acid to butanol via esterification and hydrogenolysis; and (3) syngas to butanol via thermochemical consecutive reactions.

Even though the employment of a range of heterogeneous catalysts for the conversion of ethanol to n-butanol has been extensively investigated, evaluation of all available catalytic systems that upgrade ethanol to butanol in terms of economic and environmental points of view is necessary because the technologies are able to aid in high-quality fuel production in an environmentally-friendly way. The evaluation, however, is not easily achieved on a lab scale. Therefore, the studies to evaluate catalyst performance at an industrial scale need to be carefully conducted.

Despite the high performance of the heterogeneous catalytic process to produce butanol from organic waste-derived butyric acid, durability of the catalysts at industrial scales still remains as a challenge. Although many researchers have worked to improve catalyst stability by preventing catalyst deactivation for various reactions [92], they are limited to experimental scales. Further research on improving catalyst lifetime for the ethanol production process from organic waste at large scales must be considered in order to make it more economically viable.

Societal industrial demand for the use of renewable chemicals is important to continuously develop renewable butanol production technologies. The increase in the demand requires financial benefits of capital, operation, and maintenance costs for technologies. To gain the financial benefits, the collection of biomass and organic waste should be a critical part of overall renewable butanol production cycles, and the collection cost is highly associated with legislation [93]. Hence, local systems for feedstock collection and transportation need to be improved to reduce feedstock costs, potentially gaining financial advantages. Application of government legislation further enhances the economics of the technology of renewable butanol production. The development of different process configurations with the intensification of the process would be recommended as future studies.

## Figures and Tables

**Table 1:** Catalysts used for the conversion of ethanol to n-butanol. All the results are obtained from earlier literature.

Catalyst	Reactor Type	Ethanol Phase	Reaction Conditions	Ethanol Conversion (%)	Butanol Selectivity (%)	Ref.
Hydroxyapatite (Ca/P molar ratio of 1.64)	Fixed-bed reactor	Gas phase	300 °C; 16.4 vol.% ethanol in helium; contact time of 1.78 s	14.7	76.3	[65]
Mg–Al hydrotalcite (Mg/Al molar ratio of 3)	Packed-bed reactor	Vapor phase	400 °C; 5.5 vol.% ethanol in helium (30 mL min^−1^); weight hourly space velocity (WHSV) of 0.215 h^−1^	25	16.1	[53]
Mg–Al mixed oxide (Mg/Al molar ratio of 3)	Fixed-bed reactor	Vapor phase	350 °C; 12 vol.% ethanol in N_2_ (40 mL min^−1^); 8-h reaction	~35	~38	[51]
Ni/Al_2_O_3_ (Ni loading of 20.7%)	Batch reactor	Liquid phase	250 °C; catalyst/ethanol ratio of 3.3/100 (*w*/*v*)	25	80	[55]
Cu/Al_2_O_3_, Ni/Al_2_O_3_	Fixed-bed reactor	Liquid phase	240 °C; 7 MPa (argon); liquid hourly space velocity (LHSV) of 4.3 L h^−1^; ethanol specific velocity of 8.3 × 10^−6^ m/s	20–28	60–65	[58]
Ru–bis(diphenylphosphanyl)methane	Batch reactor	Liquid phase	150 °C; Ru of 0.1 mol.%; Ru/ligand molar ratio of 1; 4 h	>20	94	[57]
Hydroxyapatite (commercial)	Fixed-bed reactor	Vapor phase	438 °C; 15.2% ethanol in argon; WHSV of 14 h^−1^	-	Yield: 15.5%	[45]
Sr_10_(PO_4_)_6_(OH)_2_	Fixed-bed reactor	Liquid phase	300 °C; 16.1 mol.% in argon; space velocity of 130 h g_cat._ mol_ethanol_^−1^; 3-h time-on-stream	20	~79	[47]
MgO	Fixed-bed reactor	Liquid phase	450 °C; 1 atm; N_2_ flow of 10 mL min^−1^	56.1	32.8	[49]
Cu–Mg–Al mixed oxide	Batch reactor	Liquid phase	200 °C; ethanol/catalyst ratio of 79; 100-h reaction	~11	~70	[54]
Pd–Mg–Al mixed oxide	Batch reactor	Liquid phase	260 °C; ethanol/catalyst ratio of 79; LHSV of 15 mL g^−1^ h^−1^; 5-h reaction	17.5	78	[66]
Ni/ZrO_2_ (Ni loading of 1 wt.%)	Fixed-bed reactor	Vapor phase	400 °C; 6.8 mol.% ethanol in N_2_; 0.52 μmol_ethanol_ m^−2^ s^−1^	7.7	12	[46]
Ni/Al_2_O_3_ (Ni loading of 8%)	Fixed-bed reactor	Liquid phase	250 °C; 17.6 MPa; WHSV of 6.4 h^−1^	35	62	[56]
Cu–Ni bimetallic catalyst	Fixed-bed reactor	Liquid phase	320 °C; 8 MPa; ethanol/catalyst ratio of 23.7; LHSV of 15 mL g^−1^ h^−1^; 18-h reaction	69.4	30.1	[28]
Cu/CeO_2_ (Cu loading of 10 wt.%)	Fixed-bed reactor	Liquid phase	260 °C; 10 MPa; ethanol/CO_2_ ratio of 0.05; LHSV of 1.97 h^−1^	67	45	[39]
Cu/CeO_2_–activated carbon (Cu/Ce = 3)	Batch reactor	Liquid phase	250 °C; 0.1 MPa N_2_; ethanol/catalyst ratio of 24.2; 48-h reaction	39.1	55.2	[42]
Ni–Mg–AlO (Ni/Mg/Al = 1/4/1)	Fixed-bed reactor	Liquid phase	250 °C; 3 MPa; N_2_ flow of 30 mL min^−1^; WHSV of 3.2 h^−1^	18.7	55.2	[41]
Pd/UiO-66 metal-organic framework (Pd loading of 2 wt.%)	Fixed-bed reactor	Liquid phase	250 °C; 2 MPa; N_2_/ethanol ratio of 250; LHSV of 4 mL g^−1^ h^−1^; 12-h reaction	49.9	50.1	[44]

**Table 2:** Catalysts used for the esterification of butyric acid to methyl butyrate. All the results are obtained from earlier literature.

Catalyst	Reaction Conditions	Methyl Butyrate Yield (%)	Ref.
Ordered mesoporous carbon (CMK-5)	360 °C; VFA/methanol = 0.5 (*v*/*v*)	~90	[76]
Carbon black	370 °C; VFA/methanol = 0.5 (*v*/*v*)	~75	[78]
Aluminium chloride hexahydrate (homogeneous)	70 °C; molar ratio of VFA/ethanol/catalyst = 1/1/0.01; 8 h	26.2	[79]
Multi-walled carbon nanotubes	360 °C; VFA/methanol = 0.5 (*v*/*v*)	90	[70]

**Table 3:** Catalysts used for the hydrogenolysis to make n-butanol. All the results are obtained from earlier literature.

Catalyst	Reactor Type	Phase	Reaction Conditions	Butanol Yield (%)	Ref.
Co/SiO_2_ (Cu loading of 5 wt.%)	Batch reactor	Liquid phase	250 °C; 5 MPa H_2_; 10 mL feed/0.8 g catalyst; 4-h reaction	19	[70]
Pt–Co/SiO_2_ (Co/Pt molar ratio of 20)	Batch reactor	Liquid phase	250 °C; 5 MPa H_2_; feed/catalyst weight ratio of 11.2; 12-h reaction	27.6	[75]
Ru–Sn/ZnO (Sn/Ru molar ratio of 2)	Fixed-bed reactor	Vapor phase	265 °C; 2.5 MPa H_2_ (130 cm^3^ min^−1^); feed rate of 0.015 mL min^−1^	>90	[80]

## Data Availability

Not applicable.
